# A Tunable Optical Bragg Grating Filter Based on the Droplet Sagging Effect on a Superhydrophobic Nanopillar Array

**DOI:** 10.3390/s19153324

**Published:** 2019-07-29

**Authors:** Meng Zhang, Jiansheng Liu, Weifeng Cheng, Jiangtao Cheng, Zheng Zheng

**Affiliations:** 1 School of Electronic and Information Engineering, Beihang University, 37 Xueyuan Rd, Beijing 100191, China; 2 Department of Mechanical Engineering, Virginia Tech, 635 Prices Fork Road, Blacksburg, VA 24061, USA

**Keywords:** droplet, nanopillars, Bragg grating, sagging, tunable filter, refractive index sensors

## Abstract

Nanostructures have been widely applied on superhydrophobic surfaces for controlling the wetting states of liquid microdroplets. Many modern optic devices including sensors are also integrated with micro- or nanostructures for function enhancement. However, it is rarely reported that both microfluidics and optics are compatibly integrated in the same nanostructures. In this paper, a novel microfluidic-controlled tunable filter composed of an array of periodic micro/nanopillars on top of a planar waveguide is proposed and numerically simulated, in which the periodic pillars endow both the Bragg grating and the superhydrophobic functions. The tunability of grating is achieved by controlling the sagging depth of a liquid droplet into the periodic pillars. Simulation results show that a narrow bandwidth of 0.4 nm and a wide wavelength tuning range over 25 nm can be achieved by such a microfluidic-based tunable optofluidic waveguide Bragg grating filter. Moreover, this proposed scheme can be easily modified as a refractive index sensor with a sensitivity of 103 nm per refractive index unit.

## 1. Introduction

Tunable optical filters have a vast variety of applications such as wavelength (de)multiplexers in optical networks, reflectors in optical switchers and tunable lasers, etc. They can be realized with many different mechanisms including whispering gallery mode (WGM) resonators [1], Mach–Zehnder interferometers [2], fiber Bragg gratings [3], Fabry–Perot microcavities [4], and waveguide Bragg gratings [5]. Among them, the waveguide Bragg grating has attracted a great deal of interest due to its easy integration with various device platforms, such as silicon-on-insulator (SOI), silica planar lightwave circuits (PLC), indium phosphide monolithic and Bloch surface wave (BSW) platforms.

The periodic structure of a waveguide Bragg grating is its most distinctive feature, where the Bragg wavelength can be modulated when the period of the structure or the refractive index of its ambient changes [6,7]. In particular, the integration of the periodic structure-based grating with a microfluidic platform can gain tunability through regulating the interfaces between a solid and liquid, liquid and gas or liquid and liquid. For example, Wu et al. recently demonstrated an adjustable filter via modulating the interfacial curvature between oil and water [8]. On the other hand, through studying the lotus leaf effect, it was found that the periodic micro-nano structures on a leaf surface are responsible for its superhydrophobility [9,10,11]. Therefore, the periodic structures, if properly designed, can lead to a substantial increase in the degree of hydrophobicity. As such, a periodically structured surface functioning synergistically as both a superhydrophobic substrate and a waveguide Bragg grating is desired. When a liquid droplet rests on the structured surface, part of the droplet may sag into the interstitial cavities of the periodic micro-nano structures. Through some kinds of external forces, such as gas pressure, electrowetting, etc., [12,13,14,15,16], the sagging depth into the cavities can be dynamically controlled, which is equivalent to modulating the refractive index of the ambient media for the waveguide Bragg grating, leading to a tunable optofluidic filter. In addition, such a tunable optofluidic filter possesses some unique features because of the liquid nature of the droplet. For example, it is easier to prepare droplets of different refractive indices with advanced properties as those commonly used refractive index matching liquids for different wavelength bands.

To achieve the optofluidic waveguide Bragg grating filter (OFWBGF) based on a superhydrophobic micro/nanopillared surface and study its influence on the performance of OFWBGF, we conducted parametric studies of the pillared surface through both theoretical analysis and eigenmode expansion (EME) simulation of the filter performance in this configuration. In this paper, the detailed design and optimization of such a tunable optofluidic filter are presented.

## 2. Working Principles and Device Design

**Figure 1:**
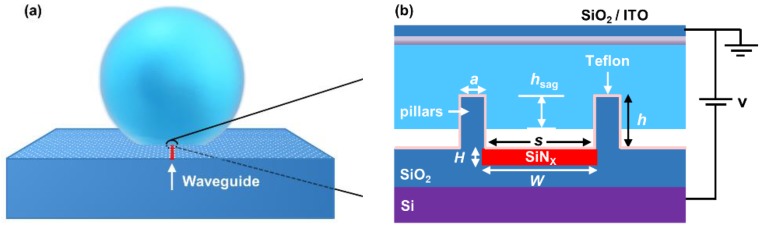
(**a**) The schematic of the proposed tunable Bragg grating filter; (**b**) The enlarged portion of the droplet–pillar–waveguide intersection. The parallel-plate electrowetting on dielectric (EWOD) is adopted in this optofluidic configuration.

Figure 1 shows the schematic of the proposed optofluidic filter. A strip waveguide is embedded in the substrate with its upper surface laying in the same plane of the substrate. On the substrate surface, an array of periodic micro/nanosized pillars is decorated to promote the superhydrophobicity of the substrate surface. In the meantime, the periodic micro/nanopillars, if properly designed, combined with the strip waveguide underneath, can also function as a waveguide Bragg grating filter. Putting a droplet on the as-formed superhydrophobic surface, the sagging of the droplet into the interstices of the periodic pillars can be precisely tuned by electrowetting on dielectric (EWOD), and the sagging depth *h*_sag_ is determined by the actuation voltage [17]. Via the EWOD actuations, the transporting speed of the three-phase contact line can reach as high as ~250 mm/s [18]. As the sagging tuning range is within hundreds of nanometers, the corresponding time of the sagging tuning and the response time of the optofluidic should be in the order of 1 μs. The EWOD mechanism can be synergized with the optical Bragg grating filter by applying voltage between the substrate and the indium tin oxide (ITO) glass on top of the droplet, as shown in Figure 1b.

### 2.1. Periodic Pillars Designed for a Superhydrophobic Surface with a Reversible Sagging Effect

**Figure 2:**
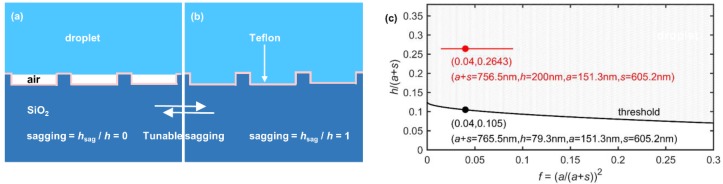
Reversible sagging effect (**a**) sagging = *h*_sag_/*h* = 0, (**b**) sagging = *h*_sag_/*h* = 1; (**c**) constraints on pillar size for a surface with θ_Y_ = 110°. Red line represents the range of pillar sizes designed for waveguide Bragg grating. Note that this range is above the threshold, which satisfies the requirement for superhydrophobicity.

Not every surface with periodic pillars would be endowed with specific wetting capabilities, e.g., superhydrophobicity, which especially enable a reversible sagging effect. Some wetting rules must be followed for designing the periodic pillars. It is well known that introducing micro- or nanoscale roughness on a solid surface can enhance the surface’s hydrophobicity to superhydrophobicity [9,10,11]. A water droplet sitting on the micro/nanostructured surface can stay either in the Cassie–Baxter state, or in the Wenzel state [19,20]. In the Cassie–Baxter state, the droplet rests atop the surface roughness and its apparent contact angle follows ${\cos\theta }_{A}=-1+f\;\left({1+\cos\theta }_{Y}\right)$, where f is the area fraction of the solid-liquid contact, 1−f is the area fraction of the solid-air contact, and $\theta_{Y}$ is the Young’s contact angle. In the Wenzel state, the droplet fully wets the substrate topography and its apparent contact angle follows ${\cos\theta }_{A}{=r}_{W}{\;\cos\theta }_{Y}$, where $r_{W}$ is the ratio of surface roughness. More recently, it has been shown that both the Cassie state (Figure 2a) and the Wenzel state (Figure 2b) are possible on the same structured surface [21], and a reversible transition between them can be achieved through applying external forces [22]. Consequently, the degree of liquid sagging into the cavities of the rough surface can be dynamically tuned.

For simplicity, square-shaped pillars are adopted in this work. Prior studies have shown that the surface structures need to satisfy certain geometric criteria to enable dynamic control of the extent of droplet sagging into the surface structures. Firstly, the height of the pillars needs to be taller than the sagging depth of the droplet. On the other hand, in order to withstand the pressure from the droplet, the density of pillars should be large enough, i.e., the period of the pillars needs to be small enough. Based on these requirements, the periodic pillars designed for a superhydrophobic surface with the reversible sagging effect must follow [23,24]:

(1) $$\frac{h}{a+s}=\frac{h}{p}\;>\;[\sqrt{2}\;-\;2\sqrt{f/\pi }]\frac{{-1+\sin\theta }_{Y}}{{2\cos\theta }_{Y}}$$

(2) $$p\;<\;2\sqrt{2}\sigma \frac{\left|{\cos(\theta }_{Y}-90{}^{\circ})\right|}{\Delta P}$$

where *h* is the height of square pillars, *a* is the pillar width, and *s* is the gap between pillars, σ = 0.0727 N/m is the surface tension of the water–air interface, ΔP is the Laplace pressure of liquid over air [23], and ${f=[a\;/\;(a+s)]}^{2}={\left(a\;/\;p\right)}^{2}$ for the square-shaped pillars with p=a+s is the period of the pillars. Their analyses indicate that for a pillared surface with Young’s contact angle *θ*_Y_, a restriction condition governed by Equations (1) and (2) for reversible Cassie–Wenzel transition exists. Figure 2c illustrates such a restriction condition for a superhydrophobic pillared surface coated by Teflon with *θ*_Y_ = 110°, in which *h*/*p* and *f* are taken as variables. The black line shows that for a given value of *f*, a threshold value of *h*/*p* exists, above which all the structure geometries are amenable to the tuning sagging effect. The pillar period *p* should be less than 1085 nm for this purpose. To achieve a large sagging-tuning range and thereafter a large wavelength-tuning range, the pillars should be designed to be higher and thinner, which corresponds to the upper-left region above the threshold line. This can be understood by the fact that higher and thinner pillars would provide a wider interstitial space for the droplet to sag into. From the point of view of optics, there would be extra requirements on the pillar geometry, which will be studied in the next section.

### 2.2. Periodic Pillars Designed for A Waveguide Bragg Grating

In order to accomplish the optical filtering functions, the periodic pillars as designed above have to be further improved to be compatible with the optic requirements of a waveguide Bragg grating at the same time. The waveguide Bragg gratings are usually constructed by introducing a periodic structure on the waveguide. Periodic structures are normally one or two dimensional micro- or nanogrooves with rectangular shapes on the top or side of the waveguide. For a waveguide Bragg grating filter, the center wavelength *λ*_B_ of the Bragg reflection is determined by the following relationship:

(3) $$\lambda_{B}=2\;\frac{p}{m}n_{\mathrm{eff}}$$

where *m* and *p* are the order and the period of the grating, respectively. *n*_eff_ is the mode effective refractive index of the grating waveguide, which can be modulated by changing the refractive index of the medium in the grooves, e.g., through tuning the droplet sagging depth in the grooves. The wavelength tuning arises from its dependence on the variation of *n*_eff_ via:

(4) $$\Delta \lambda_{B}=2\;\frac{p}{m}\Delta n_{\mathrm{eff}}$$

**Figure 3:**
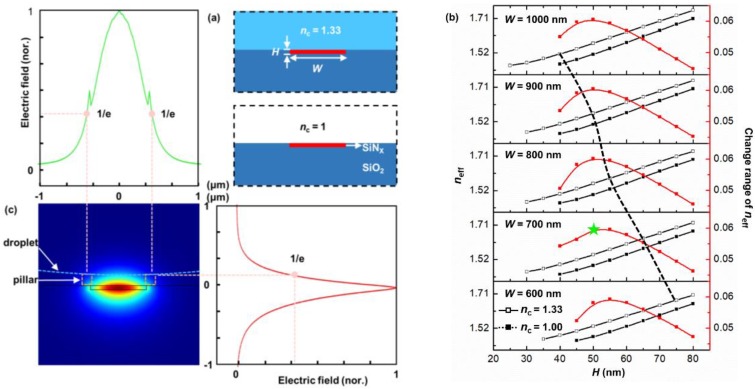
(**a**) A built-in and planar waveguide without pillars above; (**b**) Effective refractive index with respect to waveguide geometry. The dashed line is the boundary of the single mode operation; (**c**) The mode field distribution of the strip waveguide with *W* = 700 nm and *H* = 50 nm.

The proposed waveguide Bragg grating filter by us consists of a strip waveguide and a periodic pillar structure, in which the pillars, Teflon coating, air in grooves and liquid droplet function as the cladding of the waveguide in a joint manner. Regarding this tunable optofluidic filter, a narrow bandwidth can be achieved via mitigating the interaction between the evanescent field of the strip waveguide and the pillars, whereas a large wavelength tunable range can be accomplished through magnifying the interaction strength between the evanescent field of the strip waveguide and the adjustable sagging portion of the droplet. Therefore, to attain a weak interaction between the waveguide mode and the pillars but a stronger interaction between the mode and the sagging portion, the strip waveguide embedded in the square-shaped periodic nanopillars is adopted (Figure 1). On the other hand, due to the weak interaction between the mode field of the strip waveguide and the pillars, the effect of pillars can be neglected when designing and optimizing the strip waveguide (Figure 3a). For designing the strip waveguide, two extreme cases in which the strip waveguide is covered with either air (*n*_c_ = 1) or a water droplet (*n*_c_ = 1.33) are considered (Figure 3a). Here, the waveguide made of SiN_x_ embedded in the SiO_2_ substrate has a width of *W* and a height of *H*. As indicated by Equations (3) and (4), the wavelength tuning of the waveguide grating filter is determined by its effective refractive index *n*_eff_. Therefore, the effective refractive index *n*_eff_ as a function of the waveguide width *W* and height *H* was simulated by COMSOL Multiphysics, and the simulated results are shown in Figure 3b. The criteria for optimizing the geometrical parameters of the designed waveguide include three aspects. First, regardless of whether the waveguide is covered by air or water, the waveguide has to operate in the single mode for a stable operation. This requirement corresponds to the region below the black dashed line (the single mode border) in Figure 3b. Second, the difference in *n*_eff_s of the designed waveguide in the two claddings must be maximized, which infers a maximized wavelength tunable range according to Equation (4). Third, for a certain waveguide width of *W*, the range of *H* satisfying the first requirement should be large enough to relax the fabrication tolerance. By considering the aforementioned three requirements, the size of the waveguide is chosen as *W* = 700 nm and *H* = 50 nm in this work. At this optimum size of waveguide, *n*_eff_ = 1.4871 and 1.5467 in the two claddings, respectively, correspond to a large *n*_eff_ change range of 0.06. The cleanroom technology related to the fabrication of the strip SiN_x_ waveguide embedded in a substrate is quite mature. In practice, for example, the SiN_x_ layer can be first deposited on top of the SiO_2_ substrate using low temperature plasma enhanced chemical vapor deposition (PECVD), or low pressure chemical vapor deposition (LPCVD). Subsequently, the SiN_x_ strip waveguide can be patterned using conventional photolithography (mask aligner) and reactive ion etching (RIE). The fabrication of the SiO_2_ nanopillared grating can be achieved by referring to the previous reported studies [23,24].

Next, the size and period of the periodic pillars need to be scrupulously designed. According to the Bragg grating Equation (3), for a given Bragg reflection wavelength *λ*_B_, many potential gratings exist that possess different periods related by their grating order *m*. For example, if *λ*_B_ = 750 nm, the mode *n*_eff_ of the waveguide as designed above is 1.4871 when the waveguide is covered by air. Then with this value of *n*_eff_ and Equation (3), the grating periods of 252.2 nm, 756.5 nm, and 1260.8 nm can be calculated, which correspond to the grating order *m* of 1, 3 and 5, respectively. Although all of these gratings of different orders with different periods reflect coincidently at *λ*_B_ = 750 nm, the performance of their reflection spectra, such as their bandwidth and reflectivity, are quite different. Therefore, selecting an optimum grating order or grating period from these grating orders needs to be carefully addressed. As a filter, the features of a narrow bandwidth, a high reflectivity and a wide wavelength tuning range are generally expected. As such, the geometrical parameters of a grating should be meticulously designed to meet these expected performances. In order to explore the characteristics of gratings with a different order *m*, a complete waveguide grating including both the strip waveguide and the periodic pillars has to be constructed. The strip waveguide has been optimally designed as described above. For periodic pillars, the distance from the top surface of the strip waveguide vertically to the location where the intensity of the waveguide mode decays to the 1/e of its maximal value is chosen as the height of the pillars (Figure 3c). This ensures a weak but effective interaction between the mode field and the liquid droplet. From Figure 3c, it can be seen that the 1/e distance is about 110 nm, which is adopted as the pillar height in our analysis afterwards.

**Figure 4:**
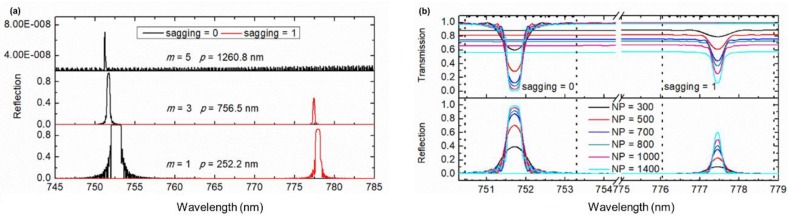
(**a**) Reflection spectra of the first, third and fifth order Bragg gratings consisting of a strip waveguide of 700 nm × 50 nm and periodic pillars of 110 nm tall with the same total grating length in the two extreme cases of sagging = 0 and sagging = 1; (**b**) Dependences of the reflection spectra and the transmission spectra on the number of period NP.

With the strip waveguide of *W* = 700 nm, *H* = 50 nm, and the periodic pillars of 110 nm tall, the spectra of the gratings with *m* = 1, 3, 5 and the same grating length of 756.5 μm were simulated, and the results are shown in Figure 4a. It can be seen that the grating with *m* = 1 possesses the largest reflectivity but the widest bandwidth, and the grating with *m* = 5 exhibits the narrowest bandwidth but a very low reflectivity. In addition, as discussed in Section 2.1, the mechanism of the reversible sagging on a superhydrophobic surface requires the periods of pillars not larger than 1085 nm. Therefore, the grating with *m* = 3 or the period *p* = 756.5 nm is the best configuration for the optical filtering purpose.

The loss *L*, i.e., the part of power that is not detected outside this structure, is also a key parameter, which is highly dependent on the length of the grating, i.e., the number of the period (NP). The loss *L* can be calculated using the relation *L* = 1 − (*T* + *R*), where *T* and *R* are the transmissivity and reflectivity, respectively. The simulated transmission and reflection spectra are shown in Figure 4b. As NP varies from 300 to 1400, i.e., corresponding to a grating length from 226.9 μm to 756.5 μm, the grating bandwidth drops quickly and tends to saturate after a long grating length, while the loss increases from 0.11 to 0.27 when the sagging = 1, and the loss increases from 0.008 to 0.01 when the sagging = 0. As a relatively small loss, a high reflectivity and a narrow bandwidth are desired; the NP is fixed at 1000, corresponding to a grating length of 756.5 μm.

**Figure 5:**
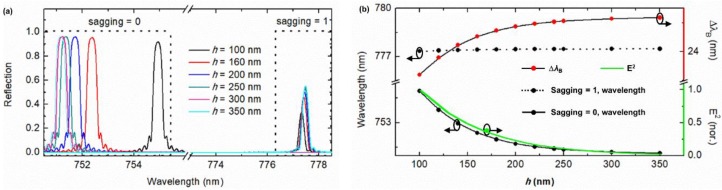
(**a**) Spectra and (**b**) *λ*_B_, ∆*λ*_B_ and mode field intensity E^2^ of the third order Bragg grating as a function of the pillar height *h*.

Although the pillar height *h* = 110 nm was utilized by us for selecting the grating order *m*, it is noteworthy that this height may be not necessarily the best option. In order to obtain the optimum pillar height, the variation of *λ*_B_ with *h* varying from 100 nm to 350 nm was numerically simulated, and the results are plotted in Figure 5. From the curves for sagging = 1 in Figure 5a,b, it can be seen that the Bragg wavelength *λ*_B_ is not sensitive to the height *h*, which infers that pillars have little effect on *λ*_B_. This fact verifies our previous assumption that the interaction of the evanescent field of the strip waveguide with the pillars is very weak, and the pillars can be neglected when designing and optimizing the strip waveguide. The curves corresponding to sagging = 0 in Figure 5a,b show that when sagging = 0, *λ*_B_ or more precisely noted as *λ*_B_ (*h*, sagging = 0) is greatly impacted by the pillar height *h* and follows a similar trend of the exponentially decaying electric field energy in the vertical direction (the green line in Figure 5b). This phenomenon coincides with the fact that the water droplet takes effect on the waveguide filter through its interaction with the evanescent field of the waveguide mode. Therefore, the variation of the interaction intensity with the pillar height *h* is proportional to the change of the evanescent field strength with the vertical distance from the waveguide surface. In addition, the dependence of ∆*λ*_B_ on *h* is dominated by *λ*_B_ (*h*, sagging = 0) due to the following relations:

(5) $$\Delta \lambda_{B}\left(h\right)=\lambda_{B}\left(h,\;\mathrm{sagging}=1\right)\;{-\;\lambda }_{B}\left(h,\;\mathrm{sagging}=0\right)\approx \mathrm{constant}-\lambda_{B}\left(h,\;\mathrm{sagging}=0\right).$$

For achieving a good balance between the wavelength tuning range ∆*λ*_B_ and the wavelength tuning sensitivity ∆[∆*λ*_B_(*h*)]/∆*h*, 200 nm is selected by us as the optimum pillar height. Furthermore, the ratio *h*/*p* ≅ 0.2643, which corresponds to the red dot in Figure 2c located in the region allowing a reversible sagging effect.

**Figure 6:**
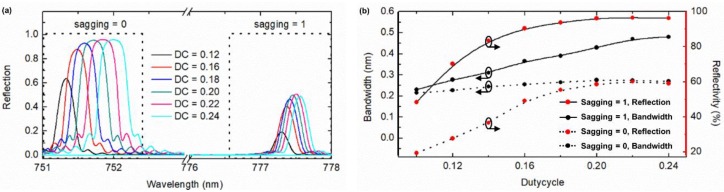
(**a**) Spectra and (**b**) bandwidth and reflectivity of the third order Bragg grating as a function of the duty cycle (*p* = 756.5 nm and *h* = 200 nm).

As another parameter of the proposed waveguide filter, the pillar width *a* also needs to be optimized, but in the form of a new parameter, i.e., duty cycle (DC) defined as DC = *a*/*p*, where *p* is fixed at the optimum value of 756.5 nm. For a given period, changing DC is equivalent to changing the interaction strength between the mode field of the waveguide and the pillars. A larger DC or pillar width corresponds to a stronger interaction, which consequently leads to a higher reflectivity but a wider bandwidth. Therefore, optimizing DC or the pillar width means balancing the bandwidth and reflectivity of the pillar-formed Bragg grating. For this purpose, the reflection spectra, the bandwidth, and the reflectivity of the Bragg grating as a function of the duty cycle DC from 0.1 to 0.24 were investigated, as shown in Figure 6. It can be seen that the reflectivity first increases and then gets saturated with increasing DC. On the other hand, the bandwidth increases with increasing DC. This trend is because the effective index modulation strength of the waveguide Bragg grating becomes higher with increasing DC. By trading off between the reflectivity and the bandwidth, the duty cycle DC = 0.2 (or the width *a* = 151.3 nm with a fixed *p* = 756.5 nm) is chosen in this work, which corresponds to *f* = 0.04 locating in the region of a superhydrophobic surface with the reversible sagging effect.

### 2.3. Wavelength Tuning by Adjusting Sagging

**Figure 7:**
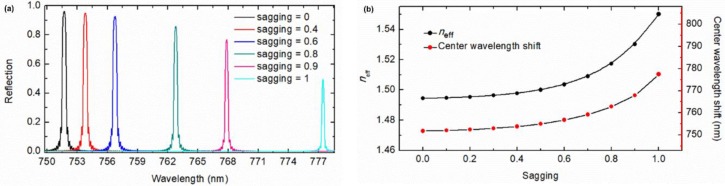
(**a**) Wavelength tuning of the Bragg grating; (**b**) Dependences of the tunable effective refractive index *n*_eff_ and the simulated center wavelength shift dependence on sagging extent *h*_sag_/*h*.

For the waveguide Bragg grating filter with the optimum waveguide size of *W* = 700 nm and *H* = 50 nm, the period *p* = 756.5 nm, the height *h* = 200 nm, and the duty cycle DC = 0.2 as designed above, its characteristics of wavelength tuning through controlling the sagging of a water droplet were investigated as plotted in Figure 7. With the variation of sagging from 0 to 1 (corresponding to the bottom of the droplet falling from the pillar top (Figure 2a) down to the grooves (Figure 2b)), the effective refractive index increases by 0.0556 (from 1.4944 to 1.55), resulting in the center Bragg wavelength shifting by 25.73 nm (from 751.71 nm to 777.45 nm). Moreover, with the same methodology, similar gratings for other wavelength bands can also be implemented by utilizing droplets of specific liquids that are transparent at the desired spectrum windows.

### 2.4. Sensing Performance

**Figure 8:**
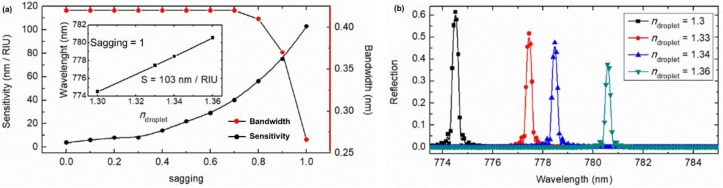
(**a**) Sensitivity and bandwidth dependence on sagging of the droplet. The inset shows the Bragg wavelength vs. *n*_droplet_; (**b**) Wavelength shift for the optofluidic filter system with sagging = 1.

Although the waveguide Bragg grating is initially designed as an optical filter, it can also be easily modified as a refractive index sensor by monitoring the wavelength shift with the change of the refractive index of the droplet. Sensitivity, the key parameter in characterizing a refractive index optical sensor, is defined as the corresponding shift rate of the wavelength [25]: ${S=d\lambda /\mathrm{dn}}_{\mathrm{droplet}}$. The sensitivities at different sagging levels were investigated. As shown in Figure 8a, a higher sensitivity is achieved at a larger sagging, and the highest S = 103 nm/RIU (refraction index unit) is reached at sagging = 1. This behavior is because a deeper sagging corresponds to a stronger interaction between the droplet and the optical field of the strip waveguide. Figure 8b displays this wavelength shift with *n*_droplet_. The bandwidth is another key parameter of optical sensors. A narrow bandwidth will result in enhanced detection resolution. The bandwidth of this optofluidic system with sagging = 1 is below 0.3 nm, which is close to that of some existing optical biosensors (e.g., bandwidth ~ 0.3 nm in a slotted photonic crystal cavity [25]). Furthermore, compared to the label-free sensing based on an EWOD waveguide [26], the center Bragg wavelength shift in the spectra of this system is preferred to intensity measurements of the waveguide. In addition, another advantage of the proposed sensor is the self-cleaning capability on the superhydrophobic surface and consequently this sensing platform can be used multiple times.

## 3. Conclusions

In this study, we exploited an optofluidic tunable filter to demonstrate the tuning capability of a waveguide Bragg grating via controlling the sagging extent of a water droplet on a pillared superhydrophobic substrate. The detailed design and optimization of the embedded strip waveguide and the pillared structures, involving the interdisciplinary photonics and microfluidics, are presented and discussed. Such a waveguide Bragg grating filter for a wavelength band of 750~780 nm is characterized with a tuning range of 25.73 nm, and a bandwidth of 0.4 nm. With the same methodology, similar gratings for other wavelength bands can also be realized by utilizing specific liquid droplets with a relevant transmission window. Furthermore, this optofluidic filter device has potential sensing applications with a high sensitivity of 103 nm/RIU.
